# Supplementary material for: Effects of intraoperative neuromonitoring (IONM) technology on early recovery quality in patients after thyroid surgery: A randomized controlled trial
Source: PLoS One. 2023 Sep 26;18(9):e0292036. doi: 10.1371/journal.pone.0292036 (PMC10522042; doi:10.1371/journal.pone.0292036)
Supplement: S1 Table — (PDF) [file pone.0292036.s001.pdf]

S1 Table. The Quality of Recovery-40 (QoR-40) Questionnaire. Positive items were scored from 1 (worst) to 5 (best); scores were reversed for negative items.

Items	Scores
**Emotional state**	
Feeling comfortable	1 (never), 2、3、4、5 (all the time)
Having a feeling of general well-being	1 (never), 2、3、4、5 (all the time)
Feeling in control	1 (never), 2、3、4、5 (all the time)
Had bad dream	1 (all the time), 2、3、4、5 (never)
Feeling anxious	1 (all the time), 2、3、4、5 (never)
Feeling angry	1 (all the time), 2、3、4、5 (never)
Feeling depressed	1 (all the time), 2、3、4、5 (never)
Feeling alone	1 (all the time), 2、3、4、5 (never)
Had difficulty falling asleep	1 (all the time), 2、3、4、5 (never)
**Physical comfort**	
Able to breathe easily	1 (never), 2、3、4、5 (all the time)
Have had a good sleep	1 (never), 2、3、4、5 (all the time)
Been able to enjoy food	1 (never), 2、3、4、5 (all the time)
Feel rested	1 (never), 2、3、4、5 (all the time)
Nausea	1 (all the time), 2、3、4、5 (never)
Vomiting	1 (all the time), 2、3、4、5 (never)
Dry-retching	1 (all the time), 2、3、4、5 (never)
Feeling restless	1 (all the time), 2、3、4、5 (never)

Shaking or twitching	1 (all the time), 2、3、4、5 (never)
Shivering	1 (all the time), 2、3、4、5 (never)
Feeling too cold	1 (all the time), 2、3、4、5 (never)
Feeling dizzy	1 (all the time), 2、3、4、5 (never)
**Psychological support**	
Able to communicate with hospital staff (during hospitalization)	1 (never), 2、3、4、5 (all the time)
Able to communicate with family or friends	1 (never), 2、3、4、5 (all the time)
Getting support from hospital doctors (when in hospital)	1 (never), 2、3、4、5 (all the time)
Getting support from hospital nurses (when in hospital)	1 (never), 2、3、4、5 (all the time)
Having support from family or friends	1 (never), 2、3、4、5 (all the time)
Able to understand instructions and advice	1 (never), 2、3、4、5 (all the time)
Feeling confused	1 (all the time), 2、3、4、5 (never)
**Physical independence**	
Able to go to the lavatory by yourself	1 (never), 2、3、4、5 (all the time)
Able to write	1 (never), 2、3、4、5 (all the time)
Have normal speech	1 (never), 2、3、4、5 (all the time)
Able to wash, brush teeth, or shave	1 (never), 2、3、4、5 (all the time)
Able to look after your own appearance	1 (never), 2、3、4、5 (all the time)
**Pain**	

**Moderate pain**	**1 (all the time), 2、3、4、5 (never)**
**Severe pain**	**1 (all the time), 2、3、4、5 (never)**
**Headache**	**1 (all the time), 2、3、4、5 (never)**
**Muscle pain**	**1 (all the time), 2、3、4、5 (never)**
**Backache**	**1 (all the time), 2、3、4、5 (never)**
**Sore throat**	**1 (all the time), 2、3、4、5 (never)**
**Sore mouth**	**1 (all the time), 2、3、4、5 (never)**
