# Supplementary material for: Effects of intraoperative neuromonitoring (IONM) technology on early recovery quality in patients after thyroid surgery: A randomized controlled trial
Source: PLoS One. 2023 Sep 26;18(9):e0292036. doi: 10.1371/journal.pone.0292036 (PMC10522042; doi:10.1371/journal.pone.0292036)
Supplement: S1 File — (PDF) [file pone.0292036.s002.pdf]

S2 File. Protocol for publication (Chinese version)

研究者发起、涉及人体的临床研究项目立项申请表

<table>
<tr><td colspan="6">项目名称：右美托咪定对甲状腺神经监测手术患者术后恢复质量的影响</td></tr>
<tr><td>本院主要研究者</td><td colspan="2">陆志俊</td><td>承担科室</td><td colspan="2">麻醉科</td></tr>
<tr><td>项目联系人</td><td colspan="2">陈浩聪</td><td>联系电话</td><td colspan="2">18018598892</td></tr>
<tr><td>研究参与人员</td><td colspan="5">陈正泽</td></tr>
<tr><td>研究设计</td><td colspan="5">☼随机 ☼对照 ☼单盲 ☐双盲 ☐开放 ☐平行 (在对应☐内打勾)
☐交叉 ☐析因 ☐成组序贯 ☐优效性 ☐非劣性 ☐其它</td></tr>
<tr><td>是否是多中心研究</td><td>☐是 ☼否</td><td>如是，牵头单位</td><td></td><td>牵头单位负责人</td><td></td></tr>
<tr><td>研究类型</td><td colspan="5">☼实验性研究 ☐观察性研究：(☐回顾性研究 ☐前瞻性研究)</td></tr>
<tr><td>本中心受试者人数/研究总人数</td><td colspan="2">90/90</td><td colspan="2">是否申请豁免知情同意</td><td>☐是 ☼否</td></tr>
<tr><td colspan="3">是否需要申请国家人类遗传资源办备案/审批</td><td colspan="3">☐是 ☼否</td></tr>
<tr><td>研究时间</td><td colspan="2">2020 年 09 月至 2021 年 04 月</td><td colspan="2">是否涉及未上市药物/医疗器械</td><td>☐是 ☼否</td></tr>
<tr><td>研究资助类型</td><td colspan="5">☐纵向课题 课题来源： 课题编号：
☐横向课题 资助方： 资助经费： 万
☼其他 ___ 自筹______</td></tr>
<tr><td colspan="6">主要研究者声明

我声明，本项目是以不损害受试者的权益为前提，探索疾病优良的诊断治疗方法或纯粹科学目的开展的临床研究，并且不接受附有影响受试者安全及公平竞争原则的资助。本项目将严格参照《瑞金医院临床研究项目管理办法（试行)》中相关规定开展临床研究。
我将遵循 GCP、方案以及伦理委员会的要求，开展本项临床研究。

签名:
日期 :</td></tr>
<tr><td colspan="6">临床科室负责人声明

我已经审查了本研究项目，我相信它是合理的。研究的设计和方法足以使研究目的得到实现，另外研究者也有适当的财力和其他方面的资源。我支持开展这项研究，因此把它递交上去，希望得到进一步审查。

签名:
日期 :</td></tr>
</table>

研究者发起、涉及人体研究项目立项材料清单

科室：麻醉科			
PI：陆志俊			
项目名称：右美托咪定对甲状腺神经监测手术患者术后恢复质量的影响			
**序号**	**立项形审材料**	**已提供**	**不适用**
1	立项申请表	☐	☐
2	研究方案（版本号：1.0 ，版本日期：2020 年 06 月 01 日）	☐	☐
3	知情同意书（版本号： 1.0 ，版本日期：2020 年 06 月 01 日， 回顾性研究可免）	☐	☐
4	豁免知情同意申请（仅适用于回顾性研究）	☐	☐
5	病例报告表（如适用，版本号： ，版本日期： 年 月 日）	☐	☐
6	研究者手册（如适用，版本号： ，版本日期： 年 月 日）	☐	☐
7	组长单位伦理批件（如适用，需盖章）	☐	☐
8	受试者样本采集、检测、保存说明（如适用）	☐	☐
9	其他（临床试验保险单、患者日记卡等，如适用）	☐	☐
10	药品说明书或医疗器械注册证（如适用）	☐	☐

签字：

日期：

上海交通大学医学院附属瑞金医院

涉及人体研究项目方案

（适用于前瞻性研究）

研究名称：	右美托咪定对甲状腺神经监测手术患者术后恢复质量的影响
方案号：	
主要研究者：	陆志俊
所属部门：	上海交通大学医学院附属瑞金医院麻醉科
起止年限：	2020 年 06 月—2021 年 04 月

上海交通大学医学院附属瑞金医院

二〇二〇 年 六 月 一 日

版本号：1.0

1. 研究摘要

1.1 摘要

**研究名称:**	右美托咪定对甲状腺神经监测手术患者术后恢复质量的影响
**研究简介：**	喉返神经损伤是甲状腺手术中最常见的并发症之一。术中喉返神经的定位与解剖是避免其发生损伤的重要途径之一。甲状腺喉返神经监测手术要求麻醉诱导时减少肌松药用量、麻醉维持时不追加肌松药，这一系列操作不但对麻醉医生的围术期麻醉管理提出了更高的要求，也可能会降低甲状腺手术神经监测患者的术后恢复质量。本研究将采用随机数字表法，将患者随机分为 3 组（每组 30 例），分别为神经监测右美干预组（A 组）、神经监测组（B组）和非神经监测组（C 组），旨在探索诱导前辅助应用右美托咪定，能否改善患者术后的恢复质量，同时也将探索右美托咪定对甲状腺手术神经监测患者插管前后血流动力学的影响，为甲状腺神经监测手术探索一套真实可行的麻醉方案。
**研究目的:**	主要研究目的：<br>探索右美托咪定能否改善甲状腺神经监测手术患者的术后恢复质量<br>次要研究目的：<br>探索右美托咪定对甲状腺手术神经监测患者插管前后血流动力学的影响
**研究对象：**	选取 2020 年 9 月至 2020 年 12 月在上海交通大学附属瑞金医院行甲状腺手术的患者。年龄在 20-60 岁；性别不限；体重 45-80kg；BMI 在 18-24 之间；ASA 分级为 I~II 级。
**研究单位/地点:**	上海交通大学附属瑞金医院
**研究干预:**	神经监测右美干预组（A 组）诱导前 10 分钟静脉滴注右美托咪定 0.6ug/kg，诱导时给予 1 倍 ED95 的罗库溴铵 0.3mg/kg，插管时给予神经监测气管导管。神经监测组（B 组）诱导前 10 分钟静脉滴注等容量生理盐水，诱导时给予 1 倍 ED95 的罗库溴铵 0.3mg/kg，插管时给予神经监测气管导管。非神经监测组（C 组）诱导前 10 分钟静脉滴注等容量生理盐水，诱导时给予 2 倍 ED95 的罗库溴铵 0.6mg/kg，插管时给予螺纹管。

研究持续时间: 2020 年 6 月至 2021 年 4 月

受试者参加时间: 从患者入手术室至患者术后 24 小时

1.2 技术路线图

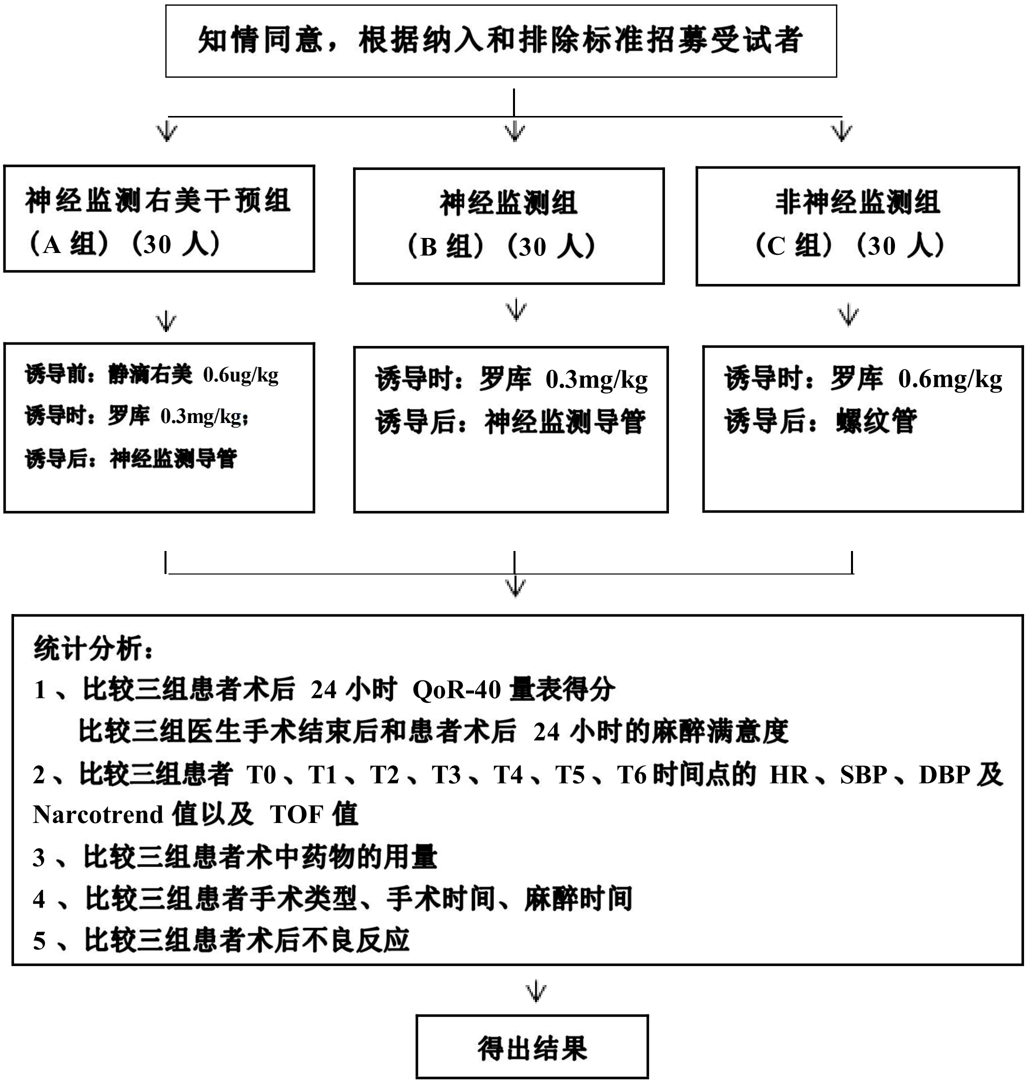

2. 研究背景

2.1 研究意义

术后恢复质量的调查目前研究较少，尤其是在甲状腺手术中。甲状腺神经监测患者术中减少肌松药用量的同时也会大大增加术中不良反应的发生，很可能会降低患者的术后恢复质量。右美托咪定能缓解患者术后疼痛、焦虑等不适，加快患者恢复，因此，本研究观察右美托咪定对甲状腺手术神经监测患者的术后恢复质量，为甲状腺神经监测手术探索一套真实可行的麻醉方案。

2.2 研究背景

喉返神经损伤是甲状腺手术中最常见的并发症之一。术中喉返神经的定位与解剖是避免其发生损伤的重要途径之一。术中神经监测（intraoperative neuromonitoring，IONM）技术近些年来广泛应用于甲状腺手术中，大量研究表明喉返神经监测可减少甲状腺手术中对喉返神经的损伤。与此同时，为了更精确的的捕捉肌电信号，外科医生也提出使用术中神经监测进行 甲状腺手术时，麻醉诱导中需要肌松药用量减半、麻醉维持过程中也不建议追加肌松药，然而这一系列的手术要求对麻醉而言无疑是一个巨大的考验和挑战，也有降低患者术后麻醉满意度的可能性。右美托咪定作为一种高选择性的α2 肾上腺素能激动剂，不但能减轻插管时的应激反应，还能缓解患者的术后疼痛，适时弥补了这一缺陷。本研究着力探索在右美托咪定的辅助下，甲状腺手术喉返神经监测患者对术后恢复质量的影响。

1、甲状腺手术喉返神经监测

喉返神经损伤是甲状腺手术最严重的并发症，即使外科操作逐渐标准化，在国内外文献报道中，喉返神经损伤的发生率仍高达 0.3%- 18.9%，且不同医生之间差别很大。近些年来， 术中神经监测技术将功能学与解剖学紧密结合，用于术中快速定位喉返神经，保护喉返神经功能完整性，降低喉返神经损伤的发生率，特别是在复杂甲状腺手术中能够提示操作风险，成为喉返神经保护的有效手段。随着监测手段的完善，喉返神经的实时监测在甲状腺手术中的应用越来越广泛。但术中喉返神经的监测对麻醉提出了新的要求，因为麻醉诱导时肌松药的使用会阻断喉返神经与相关肌肉之间的兴奋传递，影响术中对喉返神经功能的判断；然而，进行气管插管时，少用或不用肌松药又可能使患者发生呛咳反应，导致患者心率、血压甚至颅内压升高，对患者特别是有心脑疾病的患者极为不利。因此该技术对肌松药的要求非常严格，甲状腺及甲状旁腺手术中神经电生理监测临床指南中建议，术前麻醉诱导时选用中效或短效肌松药，且中效肌松药要小于常规麻醉剂量，建议选用 1 倍 ED 95 中效非去极化肌松药，术中监测结束前一般不予追加

肌松药。为了解决插管时可能出现的强烈的应激反应，麻醉诱导和维持时药物的选择对减少气管插管应激反应至关重要。

2 、右美托咪定对血流动力学的影响

右美托咪定是高选择性α2 肾上腺素能激动剂，通过减少大脑蓝斑核中的儿茶酚胺释放，产生剂量依赖性地镇静、镇痛、抗焦虑以及拟交感神经作用。有研究显示，右美托咪定能够预防和抑制全身麻醉期间的不良反应，如芬太尼类药物诱导或插管拔管时诱发的呛咳反应等。因此，右美托咪定被广泛用于减轻喉镜检查以及插管拔管带来的压力反射，特别在一些特殊插管中，如清醒气管插管中，右美托咪定被证明能够提供更好的插管条件、血流动力学稳定性以及自主呼吸的保留。甲状腺手术中应用右美托咪定辅助麻醉，有助于改善术后疼痛，减轻拔管时的应激反应，但在使用半数肌松插管方面的研究较少。本研究旨在探索，在使用半数肌松的甲状腺手术神经监测的患者中，右美托咪定对其插管前后血流动力学以及术后麻醉满意度的影响。

3 、术后恢复质量研究现状

麻醉质量是影响手术质量的主要因素，监测与提高麻醉质量对促进医疗质量的提升起着重要的作用。临床麻醉质量评价内容包括死亡率、麻醉事故发生率、麻醉纠纷、麻醉并发症的发生、麻醉失败率、麻醉满意度、术后恢复质量、疑难重症麻醉率、麻醉技术新进展等方面。Qo-R40 量表是基于患者情绪状态、身体舒适度、 自理能力、心理支持以及疼痛感受 5 个维度的综合评分， 目前被认为是评价术后恢复质量最有效且最可靠的方法。 目前，国内对术后恢复质量的研究相对较少，本研究将结合 Qo-R40 量表来探索减少肌松药用量后是否降低甲状腺手术患者的术后恢复质量以及右美托咪定是否改善甲状腺手术神经监测患者的术后恢复质量，建立一个针对甲状腺神经监测手术的术后恢复质量调查。

综上所述，甲状腺喉返神经监测手术要求麻醉诱导时减少肌松药用量、麻醉维持时不追加肌松药，这一系列操作不但对麻醉医生的围术期麻醉管理提出了更高的要求，也可能会降低甲状腺手术神经监测患者的术后恢复质量。本研究旨在探索诱导前辅助应用右美托咪定，能否改善患者术后的恢复质量，同时也将探索右美托咪定对甲状腺手术神经监测患者插管前后血流动力学的影响，为甲状腺神经监测手术探索一套真实可行的麻醉方案。

2.3 研究的预期成果

1 、应用右美托咪定后，患者术后恢复质量较高。

2 、应用右美托咪定后，医生和患者麻醉满意度较高。

3、应用右美托咪定后，患者插管前后血流动力学更稳定。

4、发表论文一篇

2.4 风险/利益评估

2.4.1 已知的潜在风险

无已知潜在风险

2.4.2 已知的潜在利益

应用右美托咪定后，患者术后恢复质量较高且患者插管前后血流动力学更加稳定。

2.4.2 潜在风险/利益评估

无潜在风险/利益评估

3. 主要研究者资料

3.1 主要研究者姓名、资格、联系方式

陆志俊，医学博士。

于 2004 年获上海交通大学医学院“百人计划”。2002 年 2 月至 6 月，在美国匹兹堡大学医学院学习肝移植麻醉。2006 年 1 月至 6 月，再次赴美进修，在美国西北大学学习危重病患者麻醉。在麻醉界具有一定知名度， 目前为上海麻醉学会青年委员，上海中西医麻醉专业委员会重症医学组成员和上海市黄浦区麻醉质控专业组副组长。同时，还担任上海交通大学医学院检验系临床医学教研室副主任。在“麻醉对记忆的影响”，“肌松药临床合理应用”和“老年人围术期心脏保护”等方面有较为深入的研究。共获得各级课题 10 项，发表国内外学术论文 40 余篇。

联系电话：13701673072

电子邮箱：lusamacn@ 163.com

3.2 主要参与人员情况

序号	姓名	性别	年龄	职称	专业	是否 GCP 培训	研究中承担的角色 (eg.PI、sub-I、CRC)
1	陈浩聪	男	26	研究生	麻醉	否	sub-PI
2	陈正泽	男	24	研究生	麻醉	否	sub-I
3							
4							
5							

4. 研究目的

主要研究目的：

探索右美托咪定能否改善甲状腺神经监测手术患者的术后恢复质量

次要研究目的：

探索右美托咪定对甲状腺手术神经监测患者插管前后血流动力学的影响

5. 研究设计

5.1 总体设计

本研究为单中心、前瞻性、随机对照试验。采用随机数字表法将患者进行随机分组。

5.2 定义研究终点

研究对象按研究方案完成了所有各阶段的研究或随访或撤回知情同意书，即到达研究终点。

5.3 确定样本量大小

根据既往文献，神经监测右美干预组 A 组术后 24 小时 Qo-R40 平均得分为 186 分，神经监测组 B 组术后 24 小时 Qo-R40 平均得分为 173 分，非神经监测 C 组术后 24 小时 Qo-R40 平均得分为 165 分， $\alpha=0.05$，$1-\beta=0.9$，运用 PASS 软件进行两两比较， 得出每组需要 25 人，加上 20-25%的剔除率和失访率，每组样本量定为 30 人

6.1 入组标准

研究对象应符合以下标准：

（1）年龄 20-60 岁；

（2）体重 45-80kg；

（3）BMI 在 18-24 之间；

（4）麻醉级别为美国麻醉医师协会（ASA）分级 I 或 II 级。

（5）行甲状腺手术的患者

6.2 排除标准

符合以下任一条标准的对象将排除于本研究:

（1）术前预测有困难气道者；

（2）对右美托咪定过敏者；

（3）未签署知情同意书；

（4）ASA ≥III 级，严重心肺疾病史，严重肝肾功能不全，严重中枢系统疾患者。

6.3 研究对象招募

2020 年 9 月-2020 年 12 月在上海交通大学附属瑞金医院行甲状腺手术的患者

6.4 研究对象分配的方法

对进行甲状腺神经监测的60 例病人进行随机分组，非神经监测组的30 例病人按照手术时间顺序入组。对进行神经监测的 60 例病人进行随机分组的方法如下：按照手术时间顺序将患者进行编号 1-60 号，用 excel 对 1-60 号产生相对应的随机数字 1’-60’，将随机数字按照从小到大的顺序进行排列，1’-30’归为神经监测右美干预组 A 组；31’-60’归为神经监测组 B 组。

7. 研究干预

7.1 给予研究干预

7.1.1 研究干预描述

神经监测右美干预组（A 组）诱导前 10 分钟静脉滴注右美托咪定 0.6ug/kg，诱导时给予 1 倍 ED95 的罗库溴铵 0.3mg/kg，插管时给予神经监测气管导管。

神经监测组（B组）诱导前 10 分钟静脉滴注等容量生理盐水，诱导时给予 1 倍 ED95 的罗库溴铵 0.3mg/kg，插管时给予神经监测气管导管。

非神经监测组（C 组），诱导前 10 分钟静脉滴注等容量生理盐水，诱导时给予 2 倍 ED95 的罗库溴铵 0.6mg/kg，插管时给予螺纹管。

7.1.2 剂量与给药方法

神经监测右美干预组（A 组）诱导前 10 分钟静脉滴注右美托咪定 0.6ug/kg 至 100ml 0.9%生理盐水中；

神经监测组（B 组）诱导前 10 分钟静脉滴注 100ml 0.9%生理盐水；

非神经监测组（C 组）诱导前 10 分钟静脉滴注 100ml 0.9%生理盐水。

7.1.3 试验药物编码的建立、保存、揭盲方法与紧急情况下破盲方法

神经监测右美干预组（A 组）采用同一批号、同一生产企业的右美托咪定（国药准字 H20183219 扬子江药业集团有限公司），对右美托咪定进行编号 1-30 以对应 A 组的 30 位患者。运用 excel

表中的随机数字表将 1-30 号对应产生相应的随机数字 1’-30’，其中编号为 1’的右美托咪定对应随机化后的 1’号患者。

7.1.4 拟进行临床、实验室检查的项目及次数

不进行临床、实验室检查

7.2 准备/处理/贮存/责任

7.2.1 责任

神经监测右美干预组（A 组）采用同一批次右美托咪定，其余两组给予同等剂量 100ml 0.9%生理盐水

7.2.2 构成、外观、包装和标签

神经监测右美干预组（A 组）采用同一批号、同一生产企业的右美托咪定（国药准字 H20183219 扬子江药业集团有限公司 ），其外观、包装都是一致的。

7.2.3 产品储藏和稳定性

A 组神经监测右美干预组中的右美托咪定进行干燥冷藏保存

7.2.4 准备

神经监测右美干预组（A 组）右美托咪定稀释到 100ml 生理盐水中。其余两组仅用 100ml 生理盐水。

7.3 缩小偏倚的措施：随机化和盲法

随机化：对进行甲状腺神经监测的60 例病人进行随机分组，非神经监测组的30 例病人按照手术时间顺序入组。对进行神经监测的 60 例病人进行随机分组的方法如下：按照手术时间顺序将患者进行编号 1-60 号，用 excel 对 1-60 号产生相对应的随机数字 1’-60’，将随机数字按照从小到大的顺序进行排列，1’-30’归为神经监测右美干预组 A 组；31’-60’归为神经监测组 B 组。

盲法：单盲，仅病人不知道入组情况

7.4 随访及依从性

充分对患者进行宣教后，将于术后 24 小时对患者进行 Qo-R40 问卷调查

7.5 研究干预承诺

认真记录患者的术后随访表，防止信息泄露

8. 研究干预中止及研究对象中止/撤出

8.1 研究干预中止

术中出现对右美托咪定过敏者的将进行干预终止

8.2 研究对象中止/撤出

在研究对象有下列情况时，研究者可以中止或撤出研究对象:

- 怀孕
- 明显的研究干预不依从
- 研究对象符合排除标准（新出现的或确认的）
- 研究对象一定时间不能接受研究干预
- 研究对象中止/撤出研究的原因应记录在病例报告表上，签署知情同意书、随机分配、但并未接受研究干预的研究对象将被代替。签署知情同意书、随机分配、接受研究干预且随后撤出的研究对象将被或不被代替。

8.3 失访

将于甲状腺手术术后 24 小时对患者进行随访，以减少失访和缺失资料的计划。

9. 研究结局评价

9.1 主要及次要结局评价

主要观察指标：

1. 比较三组患者术后 24 小时的恢复质量（QOR40 量表）

次要观察指标：

1. 比较三组医生手术结束后和患者术后 24 小时的麻醉满意度（5Likert 量表）
2. 比较三组患者的一般资料：年龄、性别、BMI 等
3. 比较三组患者药物输注前（T0）、麻醉诱导前（T1）、麻醉诱导后（T2）、插管即刻（T3）、插管后 1min（T4）、插管后 3min（T5）、插管后 5min（T6）时患者的心率（HR）、收缩压（SBP）、舒张压（DBP）、Narcotrend 值以及 TOF 值。
4. 比较三组患者术中药物的用量、术中失血量等
5. 比较三组患者手术类型、手术时间、麻醉时间、住院时间等
6. 比较三组患者术后不良反应的发生例数

9.2 安全性及其他评价

术中进行心电监护，对患者心率、血压、氧饱和度进行监测以确保手术安全

9.3 不良事件与严重不良事件

9.3.1 不良事件（AE）定义

术后出现恶心呕吐、头晕头痛、疲劳乏力等

9.3.2 严重不良事件（SAE）定义

术后出现声音嘶哑、饮水呛咳

9.3.3 不良事件分类

9.3.3.1 事件严重性

分为轻微、中度、严重

9.3.3.2 与研究干预的相关性

术后出现的恶心呕吐、头晕头痛和疲劳乏力等不良反应与右美托咪定的干预无关，与全身麻醉有关；术后出现的声音嘶哑、饮水呛咳与右美托咪定的干预无关，与手术因素有关

9.3.3.3 预期性

将对术中、术后出现的所有不良反应进行记录，必要时进行干预

9.3.4 不良事件评估的时间、频率、随访及转归

将于术后 24 小时对患者进行不良事件随访

9.3.5 不良事件报告

若出现不良反应，研究者立即向发起的人报告

9.3.6 严重不良事件报告

若出现严重不良反应，研究者立即向发起人报告，并上报严重不良反应事件

10. 统计分析

10.1 一般方法

采用 SPSS 26.0 统计软件进行分析；

1 、正态分布的计量资料采用 t 检验，组间特定时间点比较采用单因素方差分析，组内各时间点比较采用重复测量设计的方差分析；

2 、非参数资料应用 Wilcox 检验。

3 、计数资料采用卡方检验；P ＜0.05 为差异有统计学意义。

10.2 主要及次要研究终点分析

1、患者术后 24 小时的恢复质量、医生手术结束后和患者术后 24 小时的麻醉满意度采用 Student's t 检验

2 、三组患者药物输注前（T0）、麻醉诱导前（T1）、麻醉诱导后（T2）、插管即刻（T3）、插管后 1min（T4）、插管后 3min（T5）、插管后 5min（T6）时患者的心率（HR）、收缩压（SBP）、舒张压（DBP）、Narcotrend 值以及 TOF 值等，两组间比较采用 Student's t 检验，多组间两两比较采用 Student-Newman-Keuls 法进行统计分析

3 、患者术中药物的用量、术中失血量、手术时间、麻醉时间等采用 Student's t 检验

4 、患者手术类型、住院时间采用 Wilcox 检验

5 、不良反应的发生例数采用卡方检验

10.3 安全性分析

对 AE 进行编码、计算，可采用严重性、频率、与干预的关联性进行表示。导致研究干预中止的不良事件与因治疗而产生的严重 AE 应逐一列出。

10.4 基线描述性分析

采用描述性统计对各组间基线时的人口学特征与实验室指标进行比较。

10.5 亚组分析

不涉及亚组分析

11. 支持性文件与注意事项

11.1 知情同意过程

知情同意应在研究对象同意参加研究之前完成，并一直持续于整个研究过程中。知情同意书经伦理委员会同意，研究对象应阅读知情同意书。研究者会解释研究过程，并回答研究对象提出的问题；并告之研究对象可能的风险及他们的权利。研究对象在同意参与之前可与家人或监护人讨论。研究者必须告之研究对象参与研究是自愿的，并且可在研究的任何时间退出研究。知情同意书的复印件可提供给研究对象保存。研究对象的权利和福利将得到保护，并强调他们的医疗护理质量不会因为拒绝参与研究而受影响。

11.2 隐私保护

保护研究对象资料，包括相关表格、记录、样本及研究对象隐私。仅研究者能获得资料，在没有获批同意之前任何研究信息不能向未授权的第三方透露。

11.3 标本及资料的收集与使用

研究涉及的信息资料的使用，研究结束后，保留的剩余标本、影像资料及其他数据应在研究对象同意后，可用于将来的研究。

11.4 质量控制与质量保证

数据收集由临床研究人员在负责人监督下进行，负责人将对报告数据的准确性、完整性、及时性负责。所有数据应清晰以确保准确的解释，并保证其可溯源性。

11.5 数据处理与记录保存

11. 5.1 数据收集与管理

需收集患者手术时间、麻醉时间、住院时间、住院总费用、术中各特定时间点的心率、收缩压、舒张压、平均动脉压及 Nacrotrend 值。
数据收集由临床研究人员在负责人监督下进行，负责人将对报告数据的准确性、完整性、及时性负责。所有数据应清晰以确保准确的解释，并保证其可溯源性。
临床数据将建立数据库保管，数据库应有密码保护，数据库建立时应设立逻辑校对程序。

11. 5.2 研究数据保留

研究所有的数据、原始文档的保存 5 年，在销毁之前得到患者许可。

11.6 发表与数据共享约定

无数据共享约定

11.7 利益冲突声明

无利益冲突

患者知情同意书

方案名称：右美托咪定对甲状腺神经监测手术患者术后恢复质量的影响

方案编号：

知情同意书版本号：1.0，版本日期：2020 年 06 月 01 日

研究机构：上海交通大学医学院附属瑞金医院

主要研究者：陆志俊

您将被邀请参加一项临床研究。本须知提供给您一些信息以帮助您决定是否参加此项临床研究。请您仔细阅读，如有任何疑问请向负责该项研究的研究者提出。

您参加本项研究是自愿的。本次研究已通过本机构伦理委员会审查。

研究的背景和目的是什么？

喉返神经损伤是甲状腺手术中最常见的并发症之一。术中喉返神经的定位与解剖是避免其发生损伤的重要途径之一。术中神经监测（intraoperative neuromonitoring，IONM）技术近些年来广泛应用于甲状腺手术中，大量研究表明喉返神经监测可减少甲状腺手术中对喉返神经的损伤。与此同时，为了更精确的的捕捉肌电信号，外科医生也提出使用术中神经监测进行 甲状腺手术时，麻醉诱导中需要肌松药用量减半、麻醉维持过程中也不建议追加肌松药，然而这一系列的手术要求对麻醉而言无疑是一个巨大的考验和挑战，也有降低患者术后术后恢复质量的可能性。右美托咪定作为一种高选择性的α2 肾上腺素能激动剂，不但能减轻插管时的应激反应，还能缓解患者的术后疼痛，适时弥补了这一缺陷。本研究着力探索在右美托咪定的辅助下， 甲状腺手术喉返神经监测患者对术后恢复质量的影响。

如果参加研究，我需要配合做什么？

如果您同意参与这项研究，我们将对您进行编号并进行随机化分组，建立病历档案。您可能被分在 A 组右美干预组、B 组神经监测组或者 C 组非神经监测组。A 组将在麻醉诱导前 10 分钟给予右美托咪定 0.6ug/kg，诱导时给予 1 倍 ED95 的罗库溴铵 0.3mg/kg，插管时给予喉返神经监测气管导管。B 组在麻醉诱导前 10 分钟给予等容量生理盐水，诱导时给予 1 倍 ED95 的罗库溴铵 0.3mg/kg，插管时给予喉返神经监测气管导管。C 组在麻醉诱导前 10 分钟给予等容量生理盐水，诱导时给予 2 倍 ED95 的罗库溴铵 0.6mg/kg，插管时给予螺纹管。研究过程中我们需要在术后 24 小时对您进行术后恢复质量的问卷调查。您的问卷调查仅用

于研究使用。

研究结束后信息的处理：对您的问卷调查进行统计学分析

作为研究受试者，您有以下职责：提供有关自身病史和当前身体状况的真实情况；告诉研究医生自己在本次研究期间所出现的任何不适；不得服用受限制的药物、食物等；告诉研究医生自己在最近是否曾参与其他研究，或目前正参与其他研究。

研究有风险吗？

本研究的风险主要在两个方面：（1）减少肌松药用量意味着诱导时间延长，（2）右美托咪定对血流动力学有影响。

我们的应对措施：（1）在麻醉诱导时，我们有 2 位麻醉医师在为患者做辅助呼吸，确保患者在不插管的情况下不会出现缺氧；（2）我们在麻醉中会备有阿托品和麻黄素，当患者出现心率减慢，血压降低时，可以立即纠正。

我们将在术后 24 小时对您进行术后随访，问您关于术后恢复质量的看法

如果您因参与这项研究而受到伤害：如发生与该项临床研究相关的损害时，您可以获得免费治疗和/或相应的赔偿。

参与研究可能对我有什么帮助？

通过对您术后 24 小时恢复质量的问卷调查，可能为您的治疗提供必要的建议，或为疾病的研究提供有益的信息。

参与研究需要花销或有补偿吗？

费用：不需要支付任何费用

补偿：无补偿

我的信息是保密的吗？

如果您决定参加本项研究，您参加研究及在研究中的个人资料均属保密。您的问卷将以研究编号数字而非您的姓名加以标识。可以识别您身份的信息将不会透露给研究小组以外的成员，除非获得您的许可。所有的研究成员和研究申办方都被要求对您的身份保密。您的档案将保存在有锁的档案柜中，仅供研究人员查阅。为确保研究按照规定进行，必要时，政府管理部门或伦理审查委员会的成员按规定可以在研究单位查阅您的个人资料。这项研究结果

发表时，将不会披露您个人的任何资料。

我必须参加吗？

您可以选择不参加本项研究，或者在任何时候通知研究者要求退出研究，您的数据将不纳入研究结果，您的任何医疗待遇与权益不会因此而受到影响。

如果您需要其它治疗，或者您没有遵守研究计划，或者发生了与研究相关的损伤或者有任何其它原因，研究医师可以终止您继续参与本项研究。

如需要更多的信息，我应该和谁联系？

您可随时了解与本研究有关的信息资料和研究进展，若发生与本研究相关的安全性新信息，我们也会及时通知您。如果您有与本研究有关的问题，或您在研究过程中发生了任何不适与损伤，或有关于本项研究参加者权益方面的问题您可以通过 18018598892 与陈浩聪联系。

谁批准了该研究的进行？

本研究已由**上海交通大学医学院附属瑞金医院涉及人体科研伦理委员会**批准。如果您对参与本研究的权益和健康有任何问题或诉求，您可以联系本机构伦理委员会，联系电话：54661789；联系人：王译锋。

知情同意书签字页

我已经阅读了本知情同意书。

我有机会提问而且所有问题均已得到解答。

我理解参加本项研究是自愿的。

我可以选择不参加本项研究，或者在任何时候通知研究者后退出而不会遭到歧视或报复，我的任何医疗待遇与权益不会因此而受到影响。

如果我需要其它治疗，或者我没有遵守研究计划，或者发生了与研究相关的损伤或者有任何其它原因，研究医师可以终止我继续参与本项研究。

我将收到一份签过字的“知情同意书”副本。

受试者姓名：__________________________

受试者签名：___________________________

日期：________年________月________ 日

法定代理人姓名：__________________________

法定代理人签名：___________________________

日期：________年________月________ 日

见证人姓名：__________________________

见证人签名：___________________________

日期：________年________月________ 日

（注：如果受试者不识字时尚需见证人签名，如果受试者无行为能力时则需代理人签名）

我已准确地将这份文件告知受试者，要求他/她认真阅读了这份知情同意书，对所提出的问题或疑问认真解答。

研究者姓名：__________________________

研究者签名：___________________________

日期：________年________月________ 日
