# Supplementary material for: Effects of intraoperative neuromonitoring (IONM) technology on early recovery quality in patients after thyroid surgery: A randomized controlled trial
Source: PLoS One. 2023 Sep 26;18(9):e0292036. doi: 10.1371/journal.pone.0292036 (PMC10522042; doi:10.1371/journal.pone.0292036)
Supplement: S2 File — (PDF) [file pone.0292036.s003.pdf]

S3 File. Protocol for publication(English version)

Application Form for Initiation of Clinical Research Projects Initiated by Researchers Involving Human Bodies

Project name: Effect of dexmedetomidine on postoperative recovery quality of patients undergoing thyroid nerve monitoring operation					
Main researchers of the hospital	Lu Zhijun	Responsible department	Department of Anesthesiology		
Project Contact	Chen Haocong	contact number	18018598892		
Research participants	Chen Zhengze				
research design	☑ Random ☑ Control ☑ Single blind ☐ Double blind ☐ Open ☐ Parallel (tick the corresponding ☐)<br>☐ Cross ☐ Factorization ☐ Group sequential ☐ Superior ☐ Non inferior ☐ Others				
Whether it is a multi center research investigate	☐ Yes ☑ No	If yes, lead Company		Leader blame others	
Research type	☑ Experimental research ☐ Observational study: (☐ Retrospective study ☐ Prospective study)				
Number of subjects/total number of studies in the center	90/90	Whether to apply for exemption of informed consent	☐ Yes ☑ No		
Whether it is necessary to apply to the National Human Genetic Resources Office for filing/approval		☐ Yes ☑ No			
Study time	September 2020 to April 2021 month	Whether it involves unlisted drugs/medical devices	☐ Yes ☑ No		
Research funding type	☐ Longitudinal topic Topic source: Subject No.:<br>☐ Horizontal topic Sponsors: Funding: ten thousand<br>☑Others _____raise independently_____				

Statement of principal investigator

I declare that this project is a clinical research carried out for the purpose of exploring excellent diagnosis and treatment methods of diseases or purely scientific purposes on the premise of not harming the rights and interests of the subjects, and will not accept the funding attached with the principle of affecting the safety and fair competition of the subjects.This project will carry out clinical research in strict accordance with the relevant provisions of the Administrative Measures for Clinical Research Projects of Ruijin Hospital (Trial).

I will carry out this clinical study in accordance with the requirements of the GCP, the protocol and the Ethics Committee.

Signature:

Date:

Statement of head of clinical department

I have reviewed this research project and I believe it is reasonable.The design and methods of the research are enough to realize the research purpose, and the researchers also have appropriate financial resources and other resources.I support this research, so I submit it for further review.

Signature:

Date:

List of Materials for Initiation of Human Research Projects Initiated by Researchers

**Department: Anesthesia Department**			
**PI: Lu Zhijun**			
**Project name: Effect of dexmedetomidine on postoperative recovery quality of patients undergoing thyroid nerve monitoring operation**			
**S/N**	**Project approval materials**	**Provided**	**Not applicable**
1	Project Initiation Application Form	☐	☐
2	Research protocol (version No.: 1.0, version date: June 1, 2020)	☐	☐
3	Informed consent form (version number: 1.0, version date: June 1, 2020, retrospective study can Exempt)	☐	☐
4	Exemption from informed consent application (only applicable to retrospective study)	☐	☐
5	Case report form (if applicable, version number: , version date: years Day)	☐	☐
6	Investigator's Manual (if applicable, version number: , version date: years Day)	☐	☐
7	Ethics approval document of the team leader (if applicable, it needs to be sealed)	☐	☐
8	Description of sample collection, detection and storage of subjects (if applicable)	☐	☐
9	Others (clinical trial insurance policy, patient diary card, etc., if applicable)	☐	☐
10	Drug manual or medical device registration certificate (if applicable)	☐	☐

Signature:

Date:

Ruijin Hospital Affiliated to Shanghai Jiaotong University School of Medicine

(Applicable to prospective research)

Study name: Dextrmedetomidine monitoring thyroid nerve in patients undergoing surgery impact of post recovery quality

Scheme No.:

Main researchers: Lu Zhijun

Department: Department of Anesthesiology, Ruijin Hospital, Shanghai Jiaotong University School of Medicine

Starting and ending years: June 2020 April 2022

Ruijin Hospital Affiliated to Shanghai Jiaotong

University School of Medicine 2020 June 1st

Version No.: 1.0

Address: No. 197, Ruijin Second Road, Shanghai
Postal code: 200025
Tel: 0086-021-64370045-673226

1. Research summary

1.1 abstract

Study name: Effect of dexmedetomidine on postoperative recovery quality of patients undergoing thyroid nerve monitoring operation

Research introduction: Recurrent laryngeal nerve injury is one of the most common complications in thyroid surgery.The location and anatomy of recurrent laryngeal nerve during operation is one of the important ways to avoid its injury.Thyroid recurrent laryngeal nerve monitoring surgery requires reducing the amount of muscle relaxants during anesthesia induction and not adding muscle relaxants during anesthesia maintenance. This series of operations not only puts forward higher requirements for the anesthesiologist's perioperative anesthesia management, but also may reduce the quality of postoperative recovery of patients undergoing thyroid surgery with nerve monitoring.In this study, patients will be randomly divided into three groups (30 cases in each group) by using the random number table method, which are nerve monitoring dextromet intervention group (Group A), nerve monitoring group (Group B) and non nerve monitoring group (Group C). The purpose is to explore whether the auxiliary application of dextromet before induction can improve the quality of postoperative recovery of patients,At the same time, we will also explore the effect of dexmedetomidine on the hemodynamics of patients undergoing thyroid surgery with nerve monitoring before and after intubation, so as to explore a realistic and feasible anesthesia scheme for thyroid surgery with nerve monitoring.

Research purpose: Main research purposes:

To explore whether dexmedetomidine can improve the quality of postoperative recovery in patients undergoing thyroid nerve monitoring surgery

Secondary research objectives:

To explore the effect of dexmedetomidine on hemodynamics in patients undergoing thyroid surgery undergoing neuromonitoring before and after intubation

Subjects: From March, 2021 to September, 2021, the patient was selected to take the A-scan in Ruijin Hospital affiliated to Shanghai Jiaotong University.Patients with gland surgery.Aged 20-60;Gender unlimited;Weight 45-80kg;BMI between 18-24;ASA is classified as I~II.Research unit/location:

Places: Ruijin Hospital Affiliated to Shanghai Jiaotong University

Research intervention: Neuromonitoring: In the right beauty intervention group (Group A), 0.6 ug/kg of dextrometomidine was given intravenously 10 minutes before induction, 0.3 mg/kg of rocuronium was given at the time of induction, and the nerve monitoring tracheal catheter was given at the time of intubation.The nerve monitoring group (Group B)

received intravenous drip of equal volume of normal saline 10 minutes before induction, and was given 0.3 mg/kg rocuronium with one time of ED95 during induction, and the nerve monitoring tracheal catheter was given during intubation.The non nerve monitoring group (Group C) received intravenous drip of equal volume normal saline 10 minutes before induction, and was given rocuronium 0.6mg/kg twice as much as ED95 during induction, and a threaded tube was given during intubation.

Study duration: June 2020 to April 2021

Participation time of subjects: From the time when the patient enters the operating room to 24 hours after the operation

1.2 Technical roadmap

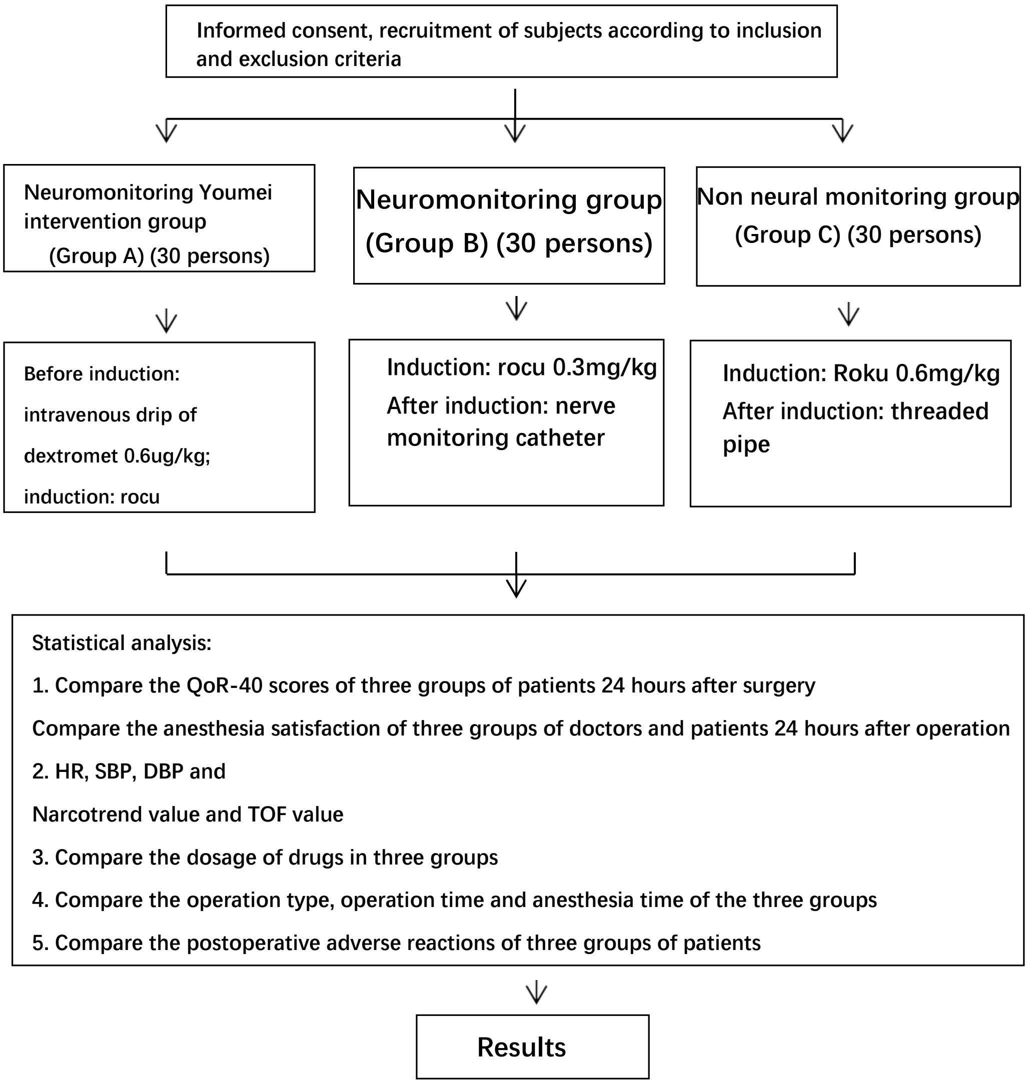

2. Research background

2.1 research meaning

At present, there is little research on the quality of postoperative recovery, especially in thyroid surgery.Reducing the amount of muscle relaxants used in thyroid nerve monitoring patients during surgery will also greatly increase the incidence of adverse reactions during surgery, which is likely to reduce the quality of postoperative recovery of patients.Dextrmedetomidine can alleviate postoperative pain, anxiety and other discomfort of patients, and speed up the recovery of patients. Therefore, this study observed the quality of postoperative recovery of patients with neuromonitoring of thyroid surgery by dexmedetomidine, and explored a set of real and feasible anesthesia scheme for thyroid neuromonitoring surgery.

2.2 Research background

Recurrent laryngeal nerve injury is one of the most common complications in thyroid surgery.The location and anatomy of recurrent laryngeal nerve during operation is one of the important ways to avoid its injury.Intraoperative neuromonitoring (IONM) technology has been widely used in thyroid surgery in recent years. A large number of studies have shown that recurrent laryngeal nerve monitoring can reduce the damage to recurrent laryngeal nerve during thyroid surgery.At the same time, in order to capture EMG signals more accurately, surgeons also proposed that when thyroid surgery was performed using intraoperative nerve monitoring, the amount of muscle relaxants needed to be halved during anesthesia induction, and it was not recommended to add muscle relaxants during anesthesia maintenance. However, this series of surgical requirements is undoubtedly a huge test and challenge for anesthesia,It is also possible to reduce patients' satisfaction with postoperative anesthesia.Dextrmedetomidine as a highly selectiveαAdrenergic agonists can not only alleviate the stress reaction during intubation, but also relieve the postoperative pain of patients, and timely make up for this defect.This study focused on exploring the effect of monitoring the recurrent laryngeal nerve on the quality of postoperative recovery in patients undergoing thyroid surgery assisted by dexmedetomidine.

1. Monitoring of recurrent laryngeal nerve in thyroid surgery

The recurrent laryngeal nerve injury is the most serious complication of thyroid surgery. Even if the surgical procedures are gradually standardized, the incidence of recurrent laryngeal nerve injury is still as high as 0.3% - 18.9% in domestic and foreign literature reports, and there is a great difference between different doctors.In recent years, intraoperative nerve monitoring technology closely combines functional and anatomical techniques to quickly locate the recurrent laryngeal nerve during operation, protect the functional integrity of the recurrent laryngeal nerve, reduce the incidence of recurrent laryngeal nerve injury, especially in complex thyroid surgery, which can prompt operational risks, and become an effective means of recurrent laryngeal nerve protection.With the improvement of monitoring means, the real-time monitoring of recurrent laryngeal nerve is more and more widely used in thyroid surgery.However, the monitoring of recurrent laryngeal nerve during operation puts forward new requirements for

anesthesia, because the use of muscle relaxants during anesthesia induction will block the excitation transmission between recurrent laryngeal nerve and related muscles, affecting the judgment of recurrent laryngeal nerve function during operation;However, when tracheal intubation is carried out, less or no muscle relaxants may cause patients to cough, which may lead to increased heart rate, blood pressure and even intracranial pressure, which is extremely unfavorable for patients, especially those with heart and brain diseases.Therefore, this technology has very strict requirements for muscle relaxants. It is suggested in the clinical guidelines for neuroelectrophysiological monitoring during thyroid and parathyroid surgery that moderate or short acting muscle relaxants should be used during preoperative anesthesia induction, and the moderate muscle relaxants should be less than the conventional anesthetic dose. It is recommended to use one time of ED 95 medium effect non depolarizing muscle relaxants, and generally no additional drugs should be added before the end of intraoperative monitoring Muscle relaxants.In order to solve the possible strong stress response during intubation, the choice of drugs during anesthesia induction and maintenance is crucial to reduce the stress response of tracheal intubation.

2. Effects of dexmedetomidine on hemodynamics

Dextrmedetomidine is highly selectiveαAdrenergic agonists produce dose-dependent sedative, analgesic, anti anxiety and sympathomimetic effects by reducing catecholamine release in the locus coeruleus nucleus.Some studies have shown that dexmedetomidine can prevent and suppress adverse reactions during general anesthesia, such as cough induced by fentanyl or induced by intubation and extubation.Therefore, dexmedetomidine is widely used to reduce the pressure reflex caused by laryngoscopy and intubation and extubation. Especially in some special intubations, such as conscious tracheal intubation, dexmedetomidine has been proved to be able to provide better intubation conditions, hemodynamic stability and reserve of spontaneous breathing.Dextrmedetomidine assisted anesthesia in thyroid surgery is helpful to improve postoperative pain and relieve stress reaction during extubation. However, there are few studies on the use of half muscle relaxant intubation.The purpose of this study was to explore the effect of dexmedetomidine on hemodynamics before and after intubation and postoperative anesthesia satisfaction in patients undergoing thyroid surgery with neuromonitoring using half of muscle relaxants.

3. Research status of postoperative recovery quality

The quality of anesthesia is the main factor affecting the quality of surgery. Monitoring and improving the quality of anesthesia play an important role in promoting the improvement of medical quality

Role of.The evaluation of clinical anesthesia quality includes mortality, incidence of anesthesia accidents, anesthesia disputes, occurrence of anesthesia complications, anesthesia failure rate, anesthesia satisfaction, quality of

postoperative recovery, rate of difficult and serious anesthesia, and new progress in anesthesia technology.Qo-R40 scale is a comprehensive score based on five dimensions of patients' emotional state, physical comfort, self-care ability, psychological support and pain perception. It is currently considered to be the most effective and reliable method to evaluate the quality of postoperative recovery.At present, there are relatively few studies on the quality of postoperative recovery in China. This study will explore whether reducing the dosage of muscle relaxants can reduce the quality of postoperative recovery in patients undergoing thyroid surgery and whether dexmedetomidine can improve the quality of postoperative recovery in patients undergoing thyroid surgery with nerve monitoring, and establish a survey on the quality of postoperative recovery in patients undergoing thyroid nerve monitoring surgery.

To sum up, the thyroid recurrent laryngeal nerve monitoring operation requires reducing the amount of muscle relaxants during anesthesia induction and not adding muscle relaxants during anesthesia maintenance. This series of operations not only puts forward higher requirements for the anesthetist's perioperative anesthesia management, but also may reduce the postoperative recovery quality of patients undergoing thyroid surgery monitoring.The purpose of this study is to explore whether the auxiliary application of dexmedetomidine before induction can improve the recovery quality of patients after surgery. At the same time, it will also explore the influence of dexmedetomidine on the hemodynamics of patients undergoing thyroid surgery who are undergoing nerve monitoring before and after intubation, so as to explore a practical anesthesia scheme for thyroid nerve monitoring surgery.

2.3 Expected results of the study

1. After the application of dexmedetomidine, the postoperative recovery quality of patients was high.
2. After dexmedetomidine was used, doctors and patients were satisfied with anesthesia.
3. After dexmedetomidine was used, the hemodynamics of patients were more stable before and after intubation.
4. Publish a paper

2.4 Risk/benefit assessment

2.4.1 Known potential risks No known potential risks

2.4.2 Known potential benefits

After dexmedetomidine was used, the postoperative recovery quality of patients was higher and the hemodynamics of patients before and after intubation was more stable.

2.4.2 Potential risk/benefit assessment No potential risk/benefit assessment

3. Information of main researchers

3.1 Name, qualification and contact information of main researcher Lu Zhijun, MD.

In 2004, he was awarded the "Hundred Talents Plan" by Shanghai Jiaotong University School of Medicine.From February to June 2002, at the University of Pittsburgh School of Medicine

Learn anesthesia for liver transplantation.From January to June 2006, he went to the United States again for further study, and studied anesthesia for critically ill patients at Northwestern University in the United States.He has a certain reputation in the anesthesia industry. Currently, he is a young member of Shanghai Anesthesia Society, a member of the Intensive Medicine Group of Shanghai Traditional and Western Medicine Anesthesia Professional Committee, and a deputy leader of the Anesthesia Quality Control Professional Group of Huangpu District, Shanghai.At the same time, he also served as the deputy director of the clinical medicine teaching and research section of the Department of Laboratory Medicine, School of Medicine, Shanghai Jiaotong University.There are more in-depth studies on "the influence of anesthesia on memory", "clinical rational use of muscle relaxants" and "perioperative cardiac protection for the elderly".A total of 10 subjects at all levels were obtained, and more than 40 domestic and foreign academic papers were published.Tel: 13701673072

mail box:lusamacn@163.com

4. Information of main researchers

4.1 Name, qualification and contact information of main researcher Lu Zhijun, MD.

In 2004, he was awarded the "Hundred Talents Plan" by Shanghai Jiaotong University School of Medicine.From February to June 2002, at the University of Pittsburgh School of Medicine

Learn anesthesia for liver transplantation.From January to June 2006, he went to the United States again for further study, and studied anesthesia for critically ill patients at Northwestern University in the United States.He has a certain reputation in the anesthesia industry. Currently, he is a young member of Shanghai Anesthesia Society, a member of the Intensive Medicine Group of Shanghai Traditional and Western Medicine Anesthesia Professional Committee, and a deputy leader of the Anesthesia Quality Control Professional Group of Huangpu District, Shanghai.At the same time, he also served as the deputy director of the clinical medicine teaching and research section of the Department of Laboratory Medicine, School of Medicine, Shanghai Jiaotong University.There are more in-depth studies on "the influence of anesthesia on memory", "clinical rational use of muscle relaxants" and "perioperative cardiac protection for the elderly".A total of 10 subjects at all levels were obtained, and more than 40 domestic and foreign academic papers were published.Tel: 13701673072

mail box:lusamacn@163.com

4.2 Main participants

4. research objective

Main research purposes:

To explore whether dexmedetomidine can improve the quality of

order number	full name	Gender	Age	title	major	GCP training or not	Role in research （eg. PI、sub-I、CRC）
1	Chen Haocong	male	26	graduate student	anaesthesia	no	sub-PI
2	Chen Zhengze	male	24	graduate student	anaesthesia	no	sub-I
3							
4							
5							

ve recovery in patients undergoing thyroid nerve monitoring surgery

Secondary research objectives:

To explore the effect of dexmedetomidine on hemodynamics in patients undergoing thyroid surgery undergoing neuromonitoring before and after intubation

5. research design

5.1 overall design

This study is a single center, prospective, randomized controlled trial.The patients were randomly divided into groups by random number table.

5.2 Define Study Endpoint

The study object reached the end point when it completed all stages of the study or follow-up or withdrew the informed consent according to the study protocol.

5.3 Determine sample size

According to previous literature, the average score of Qo-R40 24 hours after operation in group A of the right beauty intervention group of nerve monitoring was 186 points, the average score of Qo-R40 24 hours after operation in group B of nerve monitoring group was 173 points, and the average score of

Qo-R40 24 hours after operation in group C of non nerve monitoring group was 165 points,α=0.05,1-β=0.9, using PASS software to make pairwise comparison, it is concluded that 25 people are needed for each group, plus 20-25% of the rejection rate and the loss rate, the sample size of each group is set as 30 people

6.1 Inclusion criteria

The study object shall meet the following standards:

(1) Age 20-60;

(2) Weight 45-80kg;

（3） BMI between 18-24;

（4） The anesthesia level is American Society of Anesthesiologists (ASA) Grade I or Grade II.

（5） Patients undergoing thyroid surgery

6.2 Exclusion criteria

Objects meeting any of the following criteria will be excluded from this study:

（1） Preoperative prediction of difficult airway;

（2） Allergies to dexmedetomidine;

（3） Failure to sign the informed consent form;

（4） ASA ≥ Grade III, history of severe heart and lung disease, severe liver and kidney dysfunction, and severe central system disease.

6.3 Research object recruitment

Patients undergoing thyroid surgery in Ruijin Hospital affiliated to Shanghai Jiaotong University from September 2020 to December 2020

6.4 Methods of research object allocation

60 patients undergoing thyroid nerve monitoring were randomly divided into groups. 30 patients in the non nerve monitoring group were randomly divided into groups according to the operation time

Order into groups.The 60 patients undergoing nerve monitoring were randomly divided into the following methods: patients were numbered 1-60 according to the operation time sequence, and the corresponding random numbers 1 '- 60' were generated from 1-60 using excel. The random numbers were arranged from small to large, and 1 '- 30' were classified into the right beauty intervention group A of nerve monitoring;31 '- 60' were classified as Neuromonitoring Group B.

7. Research intervention

7.1 Give research intervention

7.1.1 Description of research intervention

Neuromonitoring: Dextrmedetomidine 0.6ug/kg was given intravenously 10 minutes before induction in the Youmei intervention group (Group A), and was given twice as much during induction

Rocuronium bromide of ED95 was 0.3mg/kg, and nerve monitoring tracheal tube was given during intubation.

The nerve monitoring group (Group B) received intravenous drip of equal volume of normal saline 10 minutes before induction, and was given 0.3 mg/kg rocuronium with one time of ED95 during induction, and the nerve monitoring tracheal catheter was given during intubation.

The non nerve monitoring group (Group C) received intravenous drip of equal volume normal saline 10 minutes before induction, and was given 0.6 mg/kg rocuronium twice ED95 during induction and threaded tube during intubation.

7.1.2 Dosage and administration method

Neuromonitoring: Dextrmedetomidine 0.6ug/kg to 100ml 0.9% normal saline was given intravenously 10 minutes before induction in the Youmei intervention group (Group A);

The nerve monitoring group (group B) received intravenous drip of 100ml 0.9% normal saline 10 minutes before induction;Non nerve monitoring group (group C) received intravenous drip of 100ml 0.9% normal saline 10 minutes before induction.

7.1.3 Establishment, preservation and unblinding methods of trial drug code and unblinding methods in emergency

Neuromonitoring Youmei intervention group (Group A) used the same batch number and the same manufacturer of dexmedetomidine (GYZZ H20183219)

Yangzijiang Pharmaceutical Group Co., Ltd.), numbered dexmedetomidine 1-30 to correspond to 30 patients in Group A.Use excel

The random number table in the table will generate corresponding random numbers 1 '- 30' according to numbers 1 to 30, in which dextrometomidine with number 1 'corresponds to patients with number 1' after randomization.

7.1.4 Items and times of proposed clinical and laboratory examinations will not be subject to clinical and laboratory examinations

7.2 Preparation/Handling/Storage/Responsibility

7.2.1 responsibility

Neuromonitoring: the right beauty intervention group (Group A) was given the same batch of right medetomidine, and the other two groups were given the same dose of 100ml 0.9% normal saline

7.2.2 Composition, appearance, packaging and labeling

Neuromonitoring Youmei intervention group (Group A) used the same batch number and the same manufacturer of dexmedetomidine (GYZZ H20183219)
Yangzijiang Pharmaceutical Group Co., Ltd.), its appearance and packaging are consistent.

7.2.3 Product storage and stability

Neuromonitoring in Group A Dextrmedetomidine in the Dextrmedetomidine Intervention Group was stored dry and refrigerated

7.2.4 get ready

Neuromonitoring: Dextrmedetomidine was diluted to 100ml normal saline in the right beauty intervention group

(Group A).The other two groups only used 100ml normal saline.

7.3 Measures to reduce bias: randomization and blinding

Randomization: 60 patients undergoing thyroid nerve monitoring were randomly divided into groups, and 30 patients in the non nerve monitoring group were randomly divided into groups according to their hands
The operation time was divided into groups.The 60 patients undergoing nerve monitoring were randomly divided into the following methods: patients were numbered 1-60 according to the operation time sequence, and the corresponding random numbers 1 '- 60' were generated from 1-60 using excel. The random numbers were arranged from small to large, and 1 '- 30' were classified into the right beauty intervention group A of nerve monitoring;31 '- 60' were classified as Neuromonitoring Group B.

Blind method: single blind, only patients do not know the enrollment

7.4 Follow up and compliance

Qo-R40 questionnaire survey will be conducted to patients 24 hours after surgery after full education

7.5 Research intervention commitment

Carefully record the postoperative follow-up table of patients to prevent information leakage

8. Study intervention suspension and study object suspension/withdrawal

8.1 Study intervention discontinuation

Intervention will be terminated for those who are allergic to dexmedetomidine during operation

8.2 Study object suspension/withdrawal

The investigator can suspend or withdraw the study subject in the following circumstances:

- Pregnant
- Obvious non-compliance of research intervention
- The study object meets the exclusion criteria (new or confirmed)
- The research object cannot accept the research intervention for a certain time
- The reason for the study object to suspend/withdraw from the study should be recorded on the case report

form. The study object who signed the informed consent form, randomly assigned, but did not accept the study intervention will be replaced.The subjects who signed the informed consent form, randomly assigned, accepted the research intervention and subsequently withdrew will be or will not be replaced.

8.3 Lost Visit

The patients will be followed up 24 hours after thyroid surgery to reduce the plan of missing follow-up and missing data.

9. Evaluation of research outcome

9.1 Main and secondary outcome evaluation Main observation indicators:

1. The secondary observation indicators of the quality of recovery (QOR40 scale) of the three groups of patients 24 hours after surgery were compared:

1. Compare the anesthesia satisfaction of three groups of doctors and patients 24 hours after surgery (5Likert scale)

2. Compare the general information of three groups of patients: age, gender, BMI, etc

3. The heart rate (HR), systolic blood pressure (SBP), diastolic blood pressure (DBP), Narcotrend value and TOF value of the three groups were compared before drug infusion (T0), before anesthesia induction (T1), after anesthesia induction (T2), immediately after intubation (T3), 1 min after intubation (T4), 3 min after intubation (T5), and 5 min after intubation (T6).

4. Compare the dosage of drugs and blood loss in the three groups

5. Compare the operation type, operation time, anesthesia time and hospital stay of the three groups

6. Compare the number of cases of postoperative adverse reactions in three groups

9.2 Safety and other evaluations

ECG monitoring is performed during the operation to monitor the patient's heart rate, blood pressure and oxygen saturation to ensure the safety of the operation

10. Study intervention suspension and study object suspension/withdrawal

10.1Study intervention discontinuation

Intervention will be terminated for those who are allergic to dexmedetomidine during operation

10.2Study object suspension/withdrawal

The investigator can suspend or withdraw the study subject in the following circumstances:

- Pregnant
- Obvious non-compliance of research intervention
- The study object meets the exclusion criteria (new or confirmed)
- The research object cannot accept the research intervention for a certain time

- The reason for the study object to suspend/withdraw from the study should be recorded on the case report form. The study object who signed the informed consent form, randomly assigned, but did not accept the study intervention will be replaced.The subjects who signed the informed consent form, randomly assigned, accepted the research intervention and subsequently withdrew will be or will not be replaced.

10.3Lost Visit

The patients will be followed up 24 hours after thyroid surgery to reduce the plan of missing follow-up and missing data.

11. Evaluation of research outcome

11.1Main and secondary

outcome evaluation Main

observation indicators:

1. The secondary observation indicators of the quality of recovery

(QOR40 scale) of the three groups of patients 24 hours after surgery

were compared:

7. Compare the anesthesia satisfaction of three groups of doctors and patients 24 hours after surgery (5Likert scale)

8. Compare the general information of three groups of patients: age, gender, BMI, etc

9. The heart rate (HR), systolic blood pressure (SBP), diastolic blood pressure (DBP), Narcotrend value and TOF value of the three groups were compared before drug infusion (T0), before anesthesia induction (T1), after anesthesia induction (T2), immediately after intubation (T3), 1 min after intubation (T4), 3 min after intubation (T5), and 5 min after intubation (T6).

10. Compare the dosage of drugs and blood loss in the three groups

11. Compare the operation type, operation time, anesthesia time and hospital stay of the three groups

12. Compare the number of cases of postoperative adverse reactions in three groups

11.2Safety and other evaluations

ECG monitoring is performed during the operation to monitor the patient's heart rate, blood pressure and oxygen saturation to ensure the safety of the operation

Adverse events and serious adverse events

Definition of adverse event (AE)

Postoperative nausea, vomiting, dizziness, headache, fatigue and fatigue

Definition of serious adverse event (SAE):

hoarseness and choking after operation

Classification of adverse events

Event Severity

It is divided into mild, moderate and severe

Correlation with research intervention

The adverse reactions such as nausea, vomiting, dizziness, headache, fatigue and fatigue after surgery were not related to the intervention of dexmedetomidine, but related to general anesthesia;Postoperative hoarseness and choking in drinking water have nothing to do with the intervention of dexmedetomidine, but are related to surgical factors

Anticipation

All adverse reactions during and after the operation will be recorded, and intervention will be carried out if necessary

The time, frequency, follow-up and outcome of

adverse event assessment will be followed up 24 hours

after surgery

Adverse event report

In case of adverse reaction, the investigator shall report it to the initiator immediately

Serious adverse event report

In case of serious adverse reaction, the investigator shall report to the initiator immediately and report the serious adverse reaction event

10. statistical analysis

10.1 General approach

SPSS 26.0 statistical software was used for analysis;

1. The measurement data of normal distribution were tested by t test, the specific time point comparison between groups was analyzed by one-way anova, and the time point comparison within the group was analyzed by repeated measurement design;

2. Wilcox test was used for nonparametric data.

3. Chi square test was used for counting data;$P<0.05$ means the difference is statistically significant.

10.2 Primary and secondary endpoint analysis

1. Student's t-test was used to test the recovery quality of patients 24 hours after surgery, the satisfaction of doctors and patients with anesthesia 24 hours after surgery

2. Three groups of patients: before drug infusion (T0), before anesthesia induction (T1), after anesthesia induction (T2), immediately after intubation (T3), and after intubation

Heart rate (HR), systolic blood pressure (SBP), diastolic blood pressure (DBP), Narcotrend value and TOF value of patients at 1min (T4), 3min (T5) after intubation, and 5min (T6) after intubation. Student's t-test was used for comparison between the two groups, and Student Newman Keuls method was used for statistical analysis between the two groups

3. Student's t test was used to test the amount of drugs, blood loss, operation time and anesthesia time during the operation

4. Wilcox test was used for the type of operation and length of stay

5. Chi square test is used for the number of adverse reactions

10.3 Safety analysis

AE can be coded and calculated, and can be expressed by severity, frequency and correlation with intervention.Adverse events leading to discontinuation of the study intervention and serious AEs due to treatment should be listed one by one.

10.4 Baseline descriptive analysis

Descriptive statistics were used to compare the baseline demographic characteristics and laboratory indicators between groups.

10.5 Subgroup analysis does

not involve subgroup analysis

11. Supporting documents and precautions

11.1 Informed consent process

Informed consent should be completed before the subjects agree to participate in the study and continue throughout the study.The informed consent is approved by the Ethics Committee, and the research subjects should read the informed consent.The researcher will explain the research process and answer the questions raised by the research object;And inform the research objects of the possible risks and their rights.The subjects can discuss with their families or guardians before agreeing to participate.The researcher must inform the research object that it is voluntary to participate in the study and can withdraw from the study at any time during the study.A copy of the informed consent form can be provided to the study subjects for preservation.The rights and welfare of the subjects will be protected, and the quality of their medical care will not be affected by their refusal to participate in the study.

11.2 Privacy protection

Protect the data of research objects, including relevant forms, records, samples and privacy of research objects.Only researchers can obtain data, and any research information cannot be disclosed to unauthorized third parties without approval.

11.3 Collection and use of specimens and data

The use of information materials involved in the study. After the study, the remaining specimens, image materials and other data retained should be used for future research with the consent of the study object.

11.4 Quality control and quality assurance

The data collection is carried out by the clinical researchers under the supervision of the person in charge, who will be responsible for the accuracy, integrity and timeliness of the reported data.All data shall be clear to ensure accurate interpretation and traceability.

11.5 Data processing and record keeping

11.5.1 Data collection and management

It is necessary to collect the operation time, anesthesia time, hospitalization time, total hospitalization cost, heart rate, systolic blood pressure, diastolic blood pressure, mean arterial pressure and Nacrotrend value of patients at each specific time point during the operation.

The data collection is carried out by the clinical researchers under the supervision of the person in charge, who will be responsible for the accuracy, integrity and timeliness of the reported data.All data shall be clear to ensure accurate interpretation and traceability.

The clinical data will be stored in a database, which should be password protected, and logical proofreading procedures should be set up when the database is established.

11.5.2 Retention of research data

All data and original documents of the study shall be kept for 5 years, and patient permission shall be obtained before destruction.

11.6 Publication and data

sharing agreement No data

sharing agreement

11.7 Conflict of Interest

Statement No Conflict of

Interest

Informed consent of patients

Scheme name: effect of dexmedetomidine on postoperative recovery quality of patients undergoing thyroid nerve monitoring Scheme No.:

Informed consent version number: 1.0, version date: June 1, 2020 Research institution: Ruijin Hospital affiliated to Shanghai Jiaotong University School of Medicine

Main researcher: Lu Zhijun

You will be invited to participate in a clinical study.This notice provides you with some information to help you decide whether to participate in this clinical study.Please read it carefully. If you have any questions, please ask the researcher in charge of this study.

Your participation in this study is voluntary.This research has passed the review of the Ethics Committee of the Institute.What is the background and purpose of the study?

Recurrent laryngeal nerve injury is one of the most common complications in thyroid surgery.The location and anatomy of recurrent laryngeal nerve during operation is one of the important ways to avoid its injury.Intraoperative neuromonitoring (IONM) technology has been widely used in thyroid surgery in recent years. A large number of studies have shown that recurrent laryngeal nerve monitoring can reduce the damage to recurrent laryngeal nerve during thyroid surgery.At the same time, in order to capture EMG signals more accurately, surgeons also proposed that when thyroid surgery was performed using intraoperative nerve monitoring, the amount of muscle relaxants needed to be halved during anesthesia induction, and it was not recommended to add muscle relaxants during anesthesia maintenance. However, this series of surgical requirements is undoubtedly a huge test and challenge for anesthesia,It is also possible to reduce the quality of postoperative recovery of patients.Dextrmedetomidine as a highly selectiveαAdrenergic agonists can not only alleviate the stress reaction during intubation, but also relieve the postoperative pain of patients, and timely make up for this defect.This study focused on exploring the effect of monitoring the recurrent laryngeal nerve on the quality of postoperative recovery in patients undergoing thyroid surgery assisted by dexmedetomidine.

If I participate in the research, what do I need to cooperate with?

If you agree to participate in this study, we will number and randomize you to establish medical records.You may be divided into Group A, Group B, Neuromonitoring Group, or Group C, Non Neuromonitoring Group.Group A will be given 0.6 ug/kg of dexmedetomidine 10 minutes before anesthesia induction, 0.3 mg/kg of rocuronium with one time of ED95 will be given during induction, and recurrent laryngeal nerve monitoring tracheal tube will be given during intubation.Group B was given equal volume of normal saline 10 minutes before anesthesia induction, and rocuronium 0.3mg/kg, one time of ED95, was given during induction, and recurrent laryngeal nerve monitoring tracheal catheter was given during intubation.Group C was given equal volume of normal saline 10 minutes before anesthesia induction, rocuronium 0.6mg/kg twice ED95 was given during induction, and threaded tube was given during intubation.During the study, we need to conduct a questionnaire survey on the quality of postoperative recovery 24 hours after surgery.Your questionnaire only uses

For research use.

Information processing after the study: statistical analysis of your questionnaire

As a research subject, you have the following responsibilities: provide true information about your medical history and current physical condition;Inform the study doctor of any discomfort during the study;Do not take restricted drugs, food, etc;Tell the research doctor whether he has participated in other studies recently or is currently participating in other studies.

Is research risky?

The risks of this study are mainly in two aspects: (1) reducing the dosage of muscle relaxants means prolonging the induction time, and (2) dexmedetomidine has an impact on hemodynamics.

Our countermeasures: (1) During anesthesia induction, we have two anesthesiologists who are assisting the patient with breathing to ensure that the patient will not suffer from hypoxia without intubation;(2) We will prepare atropine and ephedrine during anesthesia. When the patient's heart rate slows down and blood pressure drops, they can be corrected immediately.

We will follow up you 24 hours after the operation and ask your opinion on the quality of postoperative recovery

If you are injured due to your participation in this study: if there are damages related to this clinical study, you can get free treatment and/or corresponding compensation.

What might it help me to participate in the research?

The questionnaire survey on your recovery quality within 24 hours after surgery may provide necessary suggestions for your treatment or useful information for disease research.

Does participation in research require expense or compensation?Fee: no fee is required Compensation: no compensation

Is my information confidential?

If you decide to participate in this study, your participation in the study and personal data in the study are confidential.Your questionnaire will be identified by the study number, not your name.Information that can identify you will not be disclosed to members other than the research team unless your permission is obtained.All research members and research sponsors are required to keep your identity confidential.Your files will be kept in a locked filing cabinet for researchers only.In order to ensure that the research is carried out according to the regulations, when necessary, members of the government management department or the Ethics Review Committee can access your personal data in the research unit according to the regulations.The results of this study

At the time of publication, you will not disclose any personal information.

Do I have to attend?

You can choose not to participate in this study, or notify the researcher to withdraw from the study at any time. Your data will not be included in the study results, and your medical treatment and rights will not be affected.

If you need other treatment, or you do not comply with the research plan, or there is research related injury or any other reason, the research physician can terminate your continued participation in this study.

Who should I contact if I need more information?

You can keep abreast of the information and research progress related to this study at any time, and we will also notify you in a timely manner in case of any new safety information related to this study.If you have any questions related to this study, or you have any discomfort and injury during the study, or have questions about the rights and interests of participants in this study, you can contact Chen Haocong at 18018598892.

Who approved the study?

This study has been approved by the Human Research Ethics Committee of Ruijin Hospital affiliated to Shanghai Jiaotong University School of Medicine.If you have any questions or appeals about the rights and health of participating in this study, you can contact the Ethics Committee of this institution at 54661789;Contact: Wang Yifeng.

Bookmark page of informed consent

I have read this informed consent form.

I had the opportunity to ask questions and all of them were answered.I understand that participation in this study is voluntary.

I can choose not to participate in this study, or withdraw at any time after notifying the researcher without discrimination or retaliation, and my medical treatment and rights will not be affected.

If I need other treatment, or I do not follow the research plan, or there is research related injury or any other reason, the research physician can terminate my continued participation in this study.

I will receive a signed copy of the Informed Consent Form.

Subject name:______________________________

Signature of subject:___________________________

Date:____________year________month______day

Name of legal representative:________________________

Signature of legal representative:_______________________

Date:____________year________month______day

Name of witness:____________________________

Signature of witness:___________________________

Date:____________year________month______day

(Note: if the subject is illiterate, the witness's signature is required; if the subject is incapable of behavior, the agent's signature is required)

I have accurately informed the subject of this document and asked him/her to carefully read the informed consent form and carefully answer the questions or questions raised.

Investigator name:___________________________

Signature of investigator:________________________

Date:____________year________month______day
